# Transcriptome sequencing analysis for the identification of stable lncRNAs associated with bovine *Staphylococcus aureus* mastitis

**DOI:** 10.1186/s40104-021-00639-2

**Published:** 2021-12-13

**Authors:** Siyuan Mi, Yongjie Tang, Gerile Dari, Yuanjun Shi, Jinning Zhang, Hailiang Zhang, Xueqin Liu, Yibing Liu, Usman Tahir, Ying Yu

**Affiliations:** 1 Key Laboratory of Animal Genetics, Breeding and Reproduction, Ministry of Agriculture & National Engineering Laboratory for Animal Breeding, College of Animal Science and Technology, China Agricultural University, Beijing, 100193 China; 2 College of Veterinary Sciences and Animal Husbandry, Abdul Wali Khan University, Mardan, 23200 Pakistan

**Keywords:** Bovine mastitis, Folic acid, Long non-coding RNA, Mac-T cells, Mammary gland, *Staphylococcus aureus*

## Abstract

**Background:**

*Staphylococcus aureus* (*S. aureus*) mastitis is one of the most difficult diseases to treat in lactating dairy cows worldwide. *S. aureus* with different lineages leads to different host immune responses. Long non-coding RNAs (lncRNAs) are reported to be widely involved in the progress of inflammation. However, no research has identified stable lncRNAs among different *S. aureus* strain infections. In addition, folic acid (FA) can effectively reduce inflammation, and whether the inflammatory response caused by *S. aureus* can be reduced by FA remains to be explored.

**Methods:**

lncRNA transcripts were identified from Holstein mammary gland tissues infected with different concentrations of *S. aureus* (*in vivo*) and mammary alveolar cells (Mac-T cells, *in vitro*) challenged with different *S. aureus* strains. Differentially expressed (DE) lncRNAs were evaluated, and stable DE lncRNAs were identified *in vivo* and *in vitro*. On the basis of the gene sequence conservation and function conservation across species, key lncRNAs with the function of potentially immune regulation were retained for further analysis. The function of FA on inflammation induced by *S. aureus* challenge was also investigated. Then, the association analysis between these keys lncRNA transcripts and hematological parameters (HPs) was carried out. Lastly, the knockdown and overexpression of the important lncRNA were performed to validate the gene function on the regulation of cell immune response.

**Results:**

Linear regression analysis showed a significant correlation between the expression levels of lncRNA shared by mammary tissue and Mac-T cells (*P* < 0.001, *R*^2^ = 0.3517). lncRNAs *PRANCR* and *TNK2–AS1* could be regarded as stable markers associated with bovine *S. aureus* mastitis. Several HPs could be influenced by SNPs around lncRNAs *PRANCR* and *TNK2–AS1*. The results of gene function validation showed *PRANCR* regulates the mRNA expression of *SELPLG* and *ITGB2* within the *S. aureus* infection pathway and the Mac-T cells apoptosis. In addition, FA regulated the expression change of DE lncRNA involved in toxin metabolism and inflammation to fight against *S. aureus* infection.

**Conclusions:**

The remarkable association between SNPs around these two lncRNAs and partial HP indicates the potentially important role of *PRANCR* and *TNK2–AS1* in immune regulation. Stable DE lncRNAs *PRANCR* and *TNK2–AS1* can be regarded as potential targets for the prevention of bovine *S. aureus* mastitis. FA supplementation can reduce the negative effect of *S. aureus* challenge by regulating the expression of lncRNAs.

**Supplementary Information:**

The online version contains supplementary material available at 10.1186/s40104-021-00639-2.

## Introduction

Bovine mastitis is a tricky problem in dairy farming and leads to the decline of milk quality and remarkable economic losses worldwide [1]. Pathogen invasion to mammary gland is the main cause of this complex disease [2]. Several microorganisms, including *Escherichia coli* and *Staphylococcus aureus* (*S. aureus*), induce bovine mastitis. Mastitis induced by the contagious pathogen *S. aureus* and methicillin-resistant *S. aureus* (MRSA) is still hard to cure [3]. The host’s inflammatory response is dependent on the different lineages of *S. aureus* and the immune level of the mammary gland tissue [4–7].

Identifying stable molecular markers induced by different *S. aureus* strains can provide an effective broad-spectrum approach to the treatment and prevention of bovine *S. aureus* mastitis. Several stable markers of differentially expressed (DE) mRNAs involved in *S. aureus* infection, such as genes *SETD2*, *CYP1A1* and *SSB1*, have been identified [8]. Long non-coding RNAs (lncRNAs) are the noncoding transcripts with length longer than 200 nucleotides, widely regulate mRNA expression at the transcription level, and influence the progress of complex diseases [9, 10]. However, stable DE lncRNAs among different *S. aureus* strain infections are yet to be investigated.

The bovine mammary gland tissue (*in vivo*) and mammary gland alveolar cells (Mac-T cells, *in vitro*) are the common experimental materials for the study of *S. aureus* mastitis [8, 11]. Previous results indicated partially inconsistent bovine immune response between *in vivo* and *in vitro* results [11, 12]. Thus, under the condition of *S. aureus* infection, the combined analysis of lncRNA regulation *in vivo* and *in vitro* is also necessary for the identification of reliable lncRNA markers. Folic acid (FA), as a micronutrient, plays effective roles in reducing inflammation and mastitis incidence, and improving milk production [4, 5, 13, 14]. The remarkable effect of FA on the lncRNA expression has been widely reported [15–17]. Whether FA can regulate the inflammation induced by *S. aureus* infection by influencing lncRNA remains unknown.

In this study, *S. aureus* strains with different lineages are chosen to identify the stable DE lncRNAs of bovine *S. aureus* mastitis *in vivo* and *in vitro*. The potential interaction network among host (lncRNA and mRNA), *S. aureus* infection, and FA treatment is characterized.

## Material and methods

### Ethics statement

All procedures involving experimental animals were approved by the Animal Welfare Committee of China Agricultural University, Beijing, China. All efforts were made to minimize the suffering and discomfort of the experimental animals.

### *S. aureus* strains

Four *S. aureus* strains were used in this study. One strain was used for the individual study (*in vivo*), and the three other strains were used for the cell study (*in vitro*). All four strains were isolated from the fresh milk of Chinese Holstein cows and stored at − 80 °C. Details are shown in our previous report [18]. The strain used in the individual study was isolated from a Chinese Holstein cow with mastitis. The two strains of *S. aureus* used in the cell experiment were isolated separately from cow with low milk somatic cell counts (Strain L) and cow with mastitis symptoms (Strain M). The strain of MRSA used in the cell experiment was isolated from a cow with mastitis (Strain MM).

### Experimental design and samples information

**Fig. 1:**
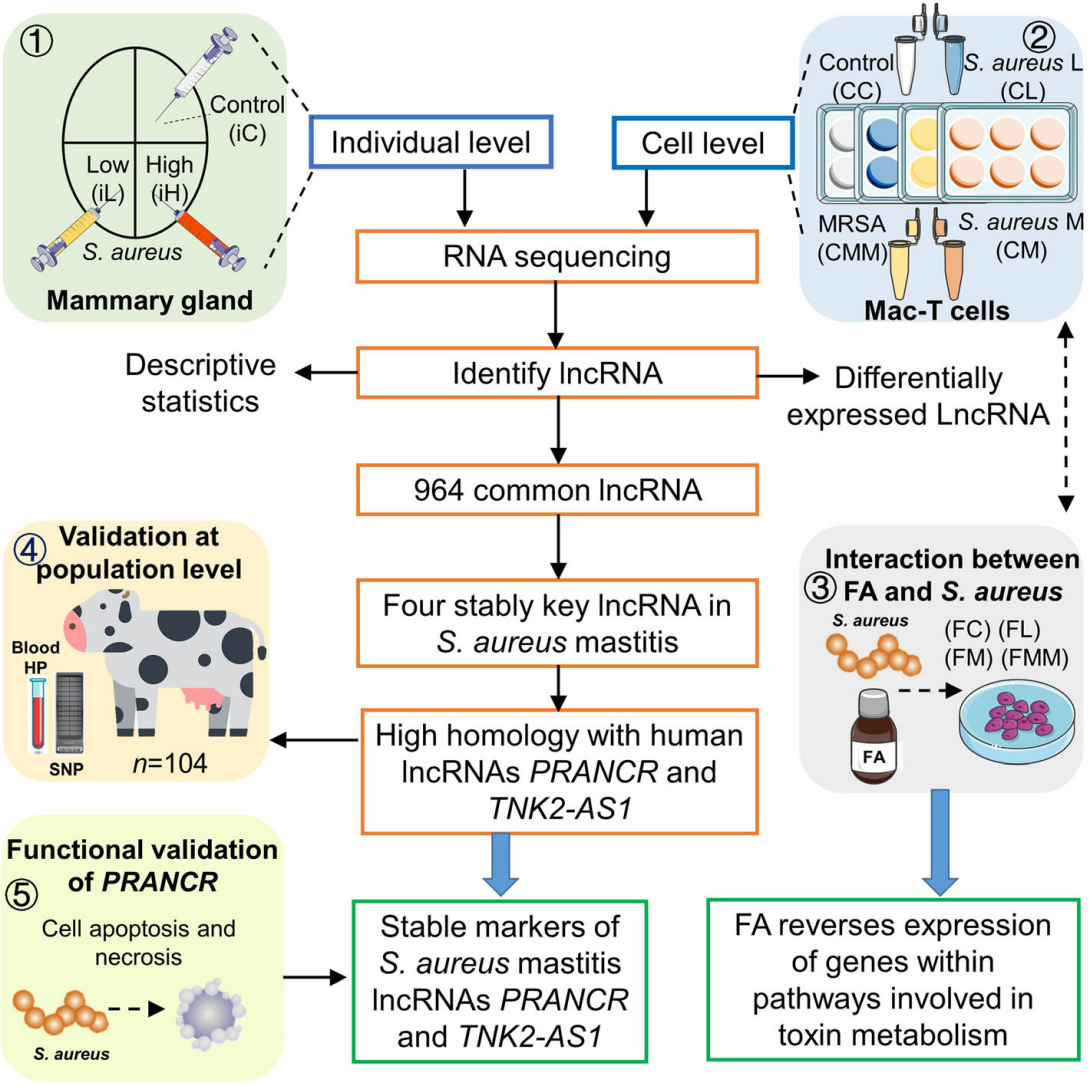
Workflow of this study. Five related experiments were designed. The bovine mammary gland was challenged with different concentrations of *S. aureus* (*in vivo*). Bovine mammary gland alveolar cells (Mac-T cells) were challenged with different *S. aureus* strains (*in vitro*). Mac-T cells were subjected to folic acid treatment and *S. aureus* challenge, and the association between SNPs around key long non-coding RNA (lncRNA) and hematological parameters (HP) was tested at the population level. Finally, the function of lncRNA was validated by gene knockdown and overexpression. *S. aureus*: *Staphylococcus aureus*; FA: folic acid; *PRANCR*: progenitor renewal associated non-coding RNA; *TNK2-AS1*: TNK2 antisense RNA 1. iC: mammary gland challenged with saline; iL: mammary challenged with low concentration of *S. aureus*; iH: mammary challenged with high concentration of *S. aureus*. CC: cells control; CL: cells challenged with Strain L; CM: cells challenged with Strain M; CMM: cells challenged with Strain MM; FC: cells treated by FA; FL: cells treated by FA and challenged with Strain L; FM: cells treated by FA and challenged with Strain M; FMM: cells treated by FA and challenged with Strain MM

Five related experiments were designed and conducted in this study to uncover the interplays among host lncRNA, *S. aureus* infection, and FA treatment (Fig. 1).

The first experiment was conducted *in vivo*. Two healthy Chinese Holstein cows during their first lactation were challenged with a strain of *S. aureus.* Details are described in our previous study [11]*.* Briefly, the three udder quarters of each cow were inoculated with 0.9% sterile pyrogen-free saline and low (10^6^ cfu/mL) and high (10^9^ cfu/mL) concentrations of the *S. aureus* strain. After the *S. aureus* challenge, udder punch biopsies were sampled from the quarters of the studied cows. In this study, mammary gland samples consisted of predominant secretory epithelial cells and a small number of other tissues, such as adipose and connective tissues. Accordingly, these groups were named as individual Control (iC), individual Low *S. aureus* (iL), and individual High *S. aureus* (iH).

The second and third experiments were finished *in vitro* by using bovine mammary alveolar cells (Mac-T cell line). Cells were treated with control, different *S. aureus* strains, or 5 μg/mL FA and infected with different *S. aureus* strains (MOI = 10:1). Eight groups (six samples per group), i.e., Control + Control (CC), Control + Strain L challenge (CL), Control + Strain M challenge (CM), Control + Strain MM challenge (CMM), FA treatment + Control (FC), FA treatment + Strain L challenge (FL), FA treatment + Strain M challenge (FM), FA treatment + Strain MM challenge (FMM), were established in the two experiments.

The fourth experiment was performed to investigate the association between bovine SNPs (in lncRNAs identified in the above experiments) and hematological parameters (HPs) of Chinese Holsteins. A total of 104 lactating Chinese Holsteins (parity ranging from 1 to 3, and lactation stage ranging from 1  to 150 d) were chosen. About 8 mL anticoagulant blood sample was collected from each cow, and 2 mL blood was sent to Jinhaikeyu Company for HP testing (Beijing, China). The genomic DNA was isolated from 400 μL blood, and SNPs were detected using the GGP Bovine HD150k (Neogen, Lansing, MI, USA).

The last experiment was the function validation of lncRNA. In this section, the knockdown and overexpression of lncRNA was carried out, and cell apoptosis and necrosis were assessed.

### Hematological parameters

Totally 24 hematological parameters were tested using the Sysmex K-4500 Automated Hematology Analyzer (Sysmex Corporation, Kobe, Japan). Specific hematological parameters are as follows: White blood cell (WBC), Red blood cell (RBC), Haemoglobin (HGB), Red blood cell specific volume (HCT), Mean Corpuscular Volume (MCV), Mean corpuscular hemoglobin (MCH), Mean corpuscular hemoglobin concentration (MCHC), Platelet count (PLT), Neutrophils ratio (NETU%), Neutrophil counts (NETU#), Lymphocyte ratio (LYMPH%), Lymphocyte counts (LYMPH#), Monocyte ratio (MONO%), Monocyte counts (MONO#), Eosinophil ratio (EO%), Eosinophil counts (EO#), Basophile ratio (BASO%), Basophile counts (BASO#), Platelet distribution width (PDW), Mean platelet volume (MPV), Red cell distribution width-stand error (RDW-SD), Red cell distribution width-coefficient of variation (RDW-CV), Platelet-large cell ratio (P-LCR), Platelet cubic measure distributing width (PCT).

### Cell culture, *S. aureus* challenge, and FA treatment

Mac-T cells were cultured in DMEM with GlutaMAX (ThermoFisher Scientific, Waltham, MA, USA) supplemented with 10% fetal bovine serum and 100 U/mL penicillin and streptomycin (ThermoFisher Scientific, Waltham, MA, USA). Initially, Mac-T cells (5 × 10^5^ cells/well) were seeded into 6-well plates. At 80% confluence, Mac-T cells were treated with DMEM with or without extra 5 μg/mL FA for 24 h and infected with three *S. aureus* isolates or control (MOI = 10:1) for 6 h. Each experimental treatment was conducted in six replicates. After the above treatments, Mac-T cells were collected and stored at − 80 °C for further RNA extraction.

### RNA extraction and sequencing

The total RNA was extracted using the TRIzol reagent (Invitrogen, Carlsbad, CA, USA) in accordance with the manufacturer’s instructions. RNA degradation was checked on 1% agarose gels. The NanoDrop 2000 (ThermoFisher Scientific, Waltham, MA, USA) was used to assess the concentration and purity of RNA. The RNA Nano 6000 Assay Kit of the Bioanalyzer 2100 system was used to measure the integrity of RNA. The average RNA integrity was more than 7 and 9 in cell and individual samples, respectively. Then, 3 μg RNA per sample was used to construct RNA-seq libraries. Finally, RNA-seq libraries were sequenced using the Illumina NovaSeq 6000 (Novogene, Beijing, China).

### Reads alignment and lncRNA identification

After the collection of RNA-seq raw data, reads alignment was performed. First, the quality of 150 bp paired-end reads were assessed using the FastQC version 0.11.8. Clean reads were obtained using the Trimmomatic software version 0.38 with default parameters (http://www.usadellab.org/cms/?page=trimmomatic). Clean reads were mapped to the bovine reference genome ARS-UCD1.2 (Ensembl annotation release 98) by using the HISAT2 version 2.1.0. After sorting and indexing using the Samtools version 1.9, each sample was assembled with the StringTie version 1.3.5. All assembled transcripts were merged into a new annotation file (GTF format) by using the GffCompare.

Novel lncRNA transcripts were identified in accordance with the following criteria. First, transcripts with length ≥ 200 nt and exon number ≥ 2 were retained. Among the different classes of the GffCompare, only class codes annotated by “i” (intronic lncRNA), “u” (intervening noncoding RNA), and “x” (antisense lncRNA) were retained, and the class code of known lncRNA transcripts was annotated by “=”. Finally, four software programs (i.e., CNCI, CPAT, CPC2, and PLEK) were applied to protein-coding potential prediction about transcripts annotated by “i”, “u”, and “x”, and transcripts with CNCI score < 0, CPAT score < 0.364, CPC2 score < 0, and PLEK score < 0 were retained. In this study, transcripts that met the above criteria were regarded as lncRNA transcripts. The FeatureCounts quantified the transcript abundance under the default setting, and the read count was normalized using the DESeq2.

### Prediction of the *cis* and *trans* target mRNAs of lncRNA

In this study, mRNAs within 100 kb upstream and downstream of a lncRNA were defined as the *cis* target mRNA of the lncRNA, and this step was performed using the Bedtools. mRNA that had significant associations (|Pearson correlation| > 0.90 and *P* < 0.05) between itself and lncRNA expression was defined as the potential *trans* target mRNA of the lncRNA.

### Differentially expressed gene identification and functional enrichment analysis

The differential expressed (DE) gene analysis was performed using the DESeq2 package in R. The read count from the FeatureCounts was normalized, and the rlog-normalized read count was also calculated. The normalized read count was then used to perform the differential expression analysis, and the rlog-normalized read count was used to conduct the multidimensional scaling.

DE genes with different criteria were defined in this study. The DE lncRNA of individual samples followed the criteria of *P* < 0.05 and |log_2_fold change| > 1, and the DE lncRNA of cell samples followed the criteria of *q* < 0.05 and |log_2_fold change| > 1.

In the integrated analysis of lncRNAs identified in individual and cell samples, lncRNA with the sum of corrected read counts in all individual samples > 3 and lncRNA with the sum of corrected read counts in all cell samples > 12 were retained to exclude the random error caused by low abundance lncRNA in this section. In addition, owing to the absence of intersection under the above filtering criteria (*q* < 0.05 and |log_2_Fold change| > 1) of DE lncRNA, lncRNAs with criteria *P* < 0.05 and same expression change direction in individual and cell *S. aureus* infection treatments were defined as stable DE lncRNAs for *S. aureus* infection treatment.

The Kyoto Encyclopedia of Genes and Genomes (KEGG) enrichment analysis was performed using the target mRNAs of lncRNAs in the WebGestalt (http://www.webgestalt.org/). For the identification of quantitative trait loci (QTLs) around DE lncRNAs, cattle QTLs were available to the AnimalQTLdb (https://www.animalgenome.org/cgi-bin/QTLdb/index), and QTLs within 100 kb upstream and downstream of lncRNAs were retained.

### Plasmid and shRNA transfection

Plasmid and shRNA construction were supported by Hitrobio.tech (Beijing, China). Full-length sequences of bovine lncRNA *PRANCR* were commercially synthesized and cloned into the pcDNA3.1 + vector. pcDNA3.1 + was used as vector control for analysis. shRNA targeting sequences are listed as follows: *PRANCR* (bovine), 5′-GGTGCTTGTGCACGCACTTCC-3′ and Scramble control sequence, 5′-GTCTCCGAACGTGTCACGT-3′. shRNA targeting sequences were cloned into pLKO.1-Puro. Plasmids and shRNAs were transfected into cells by using the Entranster-H4000 (Engreen Biosystem, Beijing, China) following the manufacturer’s recommended procedures.

### Real-time PCR

The total RNA was extracted from the target cell by using the Trizol reagent (Invitrogen, Carlsbad, CA, USA) in accordance with the manufacturer’s recommendation. Then, 1 μg RNA was reverse-transcribed using the RT reagent Kit (Takara, Shiga, Japan). The mRNA expression level was determined through qRT-PCR by using the SYBR Green I Master mix (Roche, Basel, Swiss) and analyzed on the Roche LightCycler 480 instrument. The *GAPDH* gene was used as the reference gene of target gene expression. The 2^−ΔΔCt^ method was used to calculate the relative gene expression level. Primers are listed as follows:

*GAPDH*-Forward: 5′-GGTGCTGAGTATGTGGTGGA-3′, *GAPDH*-Reverse: 5′-GGCATTGCTGACAATCTTGA-3′, *PRANCR*-Forward: 5′-TCTGCTCCCTGAAACGCATC-3′, *PRANCR*-Reverse: 5′- TACCAACGGTTTCGGCTGAC-3′, *SELPLG*-Forward: 5′- CTGAGCACGGTGCCATGTTTC-3′, *SELPLG*-Reverse: 5′-CTCTGGAGGGTCCGTTTGTC-3′, *ITGB2*-Forward: 5′- GAGTGCGACAACGTCAACTG-3′, and *ITGB2*-Reverse: 5′- ATGCCGAACCCTCATACTGC-3′.

### Apoptosis and necrosis detection

A total of 5 × 10^4^ cells within 0.1 mL complete medium per well were seeded into 96-well plates. When the logarithmic stage was reached, 0.1 μg/well plasmid or shRNA was transfected into cells for 36 h. Then cells were challenged with *S. aureus* solution for 6 h at an MOI of 10:1. Afterward, the Apoptosis and Necrosis Detection Kit (Beyotime, Shanghai, China) was used to determine cell apoptosis and necrosis. All determination steps were performed following the manufacturer’s instructions. Finally, the fluorescence value was determined using the SpectraMax i3x Multi-Mode Microplate Reader (Molecular Devices, San Jose, CA, USA).

For fluorescence staining, a total of 2.5 × 10^5^ cells within 0.5 mL of complete medium per well were seeded into 24-well plates. Similarly, after transfection and *S. aureus* challenge, cell apoptosis was determined by the above Apoptosis and Necrosis Detection Kit. Samples were photographed by Nikon ECLIPSE Ts2 fluorescence microscope (Nikon, Tokyo, Japan). Apoptotic cells were stained green by YO-PRO-1. In this section, Mac-T cells were challenged with the representative *S. aureus* strain Newman.

### Statistical analysis

The linear regression analysis was performed using the GraphPad Prism. Significant differences between treatment and control groups were examined using the Student’s *t*-test.

The general linear model procedure was used to detect the effects of polymorphism on hematological parameters (HPs) by using the Statistic Analysis System Version 9.2. Ten SNPs within 100 kb of bovine lncRNA *PRANCR* (Chromosome 15: 43,166,523 ~ 43,368,050) and eight SNPs within 100 kb of bovine lncRNA *TNK2–AS1* (Chromosome 1: 70,540,306 ~ 70,741,382) were analyzed. Multiple tests were performed using the Bonferroni *t* method. In this study, *P*-value less than 0.05 indicated a significant difference. The effects included in the model were the same for all HPs:

$$ {y}_{ijkl}=\mu +{P}_i+{LS}_j+{SNP}_k+{e}_{ijkl}, $$

where *y*_*ijkl*_ is the measure for HPs, *μ* is the overall mean, *P*_*i*_ is the fixed effect of parity (*i* = 1, 2, and 3, representing parities 1, 2, and 3, respectively), *LS*_*j*_ is the fixed effect of lactation stage (*j* = 1, 2, and 3, representing lactation stages 1–50, 51–100, and 101–150 d, respectively), *SNP*_*k*_ is the fixed effect of polymorphism for each SNP, and *e*_*ijkl*_ is the residual effects.

## Results

### Overview of high-throughput sequencing data in individual and cell samples

In summary, 6 samples *in vivo* and 48 samples *in vitro* were used for the RNA-seq analysis (Fig. 1). After quality trimming, 161 million and 1122 million clean read pairs were obtained from all cDNA libraries that included 6 individual samples from cow mammary gland tissue and 48 cell samples from bovine mammary gland alveolar cells (Mac-T cells), respectively. On average, 86.02% and 97.24% of the reads from individual and cell samples, respectively, were mapped to the cattle genome (Version: ARS-UCD1.2) by using HISAT2. Of these, 81.50% and 91.85% were uniquely mapped reads, and 4.07% and 2.19% were multi-mapped reads (Table 1).

**Table 1:** Summary of reads mapped to bovine genome

Items	Cell (Mac-T cell)	Individual (Mammary gland tissue)
Clean reads	1,122,383,385	161,180,893
Total mapped reads	1,094,511,424	138,645,272
Total mapped rate, %	97.52	86.02
Unique mapping reads	1,030,932,323	131,362,428
Multi-mapping rate, %	2.19	4.07
Unique mapping rate, %	91.85	81.50

### Identification and characterization of lncRNAs in individual and cell samples

**Fig. 2:**
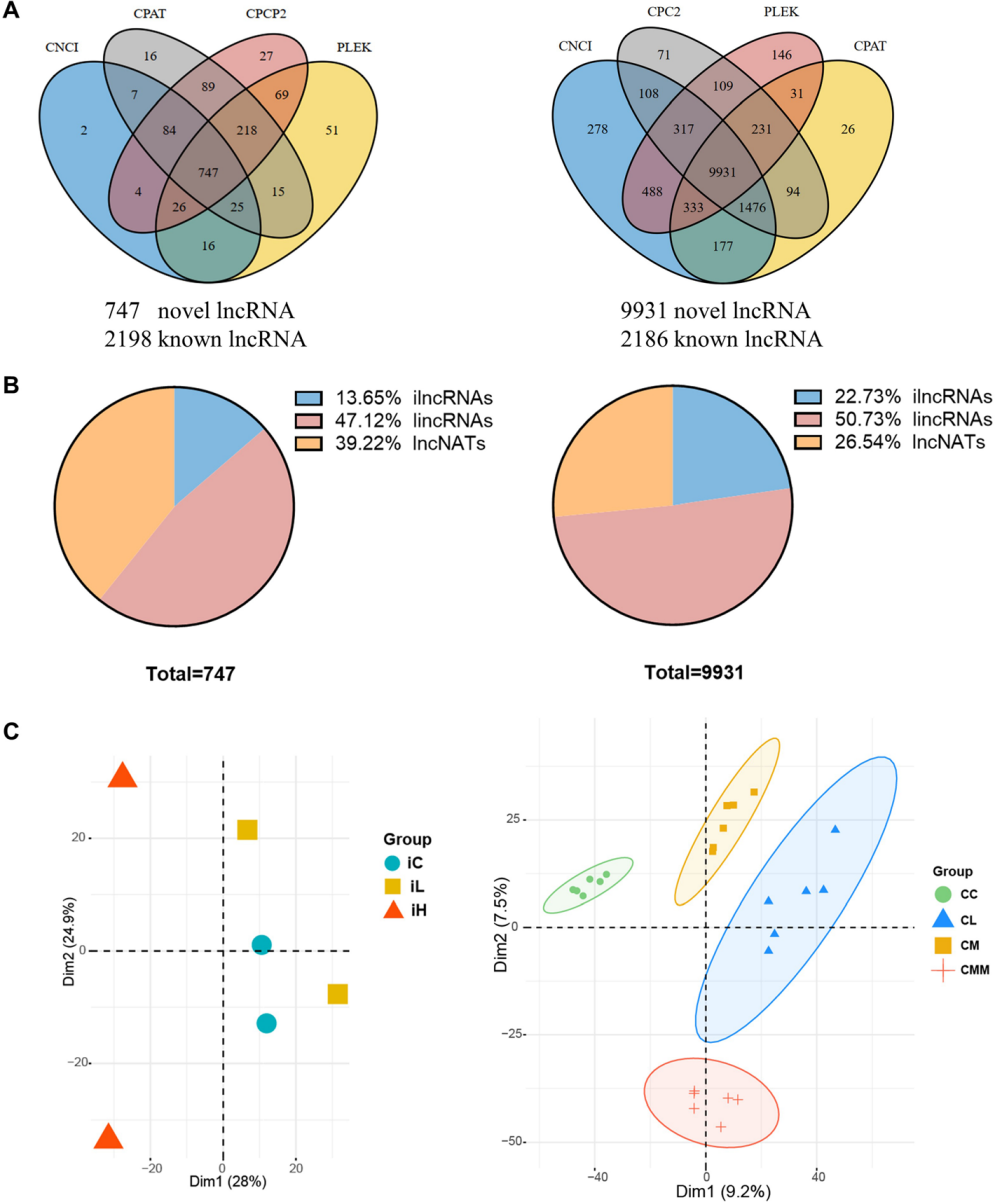
Identification of lncRNAs and principal component analysis in individual and cell samples. (**A**) Venn diagrams for the prediction of potentially novel lncRNAs. CNCI, CPAT, CPC2, and PLEK software programs were applied to protein-coding potential prediction. (**B**) Category of novel identified lncRNAs. (**C**) Multidimensional scaling of different groups. Individual and cell samples are shown on the left and right panels, respectively. ilncRNAs: intronic lncRNAs, lincRNAs: intervening noncoding RNAs, lncNATs: antisense lncRNAs

A series of filter criteria was used to determine novel lncRNA candidates. First, 68,094 and 136,375 assembled transcripts were obtained using the StringTie in individual and cell samples, respectively. After transcript filtration and protein-coding potential prediction, 747 and 9931 non-coding sequences were obtained in individual and cell samples, respectively, which were considered as novel lncRNA transcripts. In summary, 2945 (2198 known) and 12,117 (2186 known) lncRNA transcripts were obtained in individual (left panel, Fig. 2A) and cell samples (right panel, Fig. 2A), respectively. Among the 747 and 9931 identified lncRNA transcripts, 102 and 2257, respectively, were intronic lncRNAs (ilncRNAs); 352 and 5038, respectively, were intervening noncoding RNAs (lincRNAs), and 293 and 2636, respectively, were antisense lncRNAs (lncNATs) in individual and cell samples (Fig. 2B). The proportions of ilncRNAs (13.65% and 22.73%) and lncNATs (39.22% and 26.54%) in individual and cell samples were remarkably different. The multidimensional scaling was conducted to examine the intragroup consistency and intergroup specificity, and the results showed that high specificity among different groups in individual and cell samples (Fig. 2C). In addition, cell samples with or without *S. aureus* infection could distinguish control and *S. aureus* infection groups at dim1, and samples with or without MRSA infection could be regarded as a vital influence factor at dim2, which was consistent with the experiment design.

**Fig. 3:**
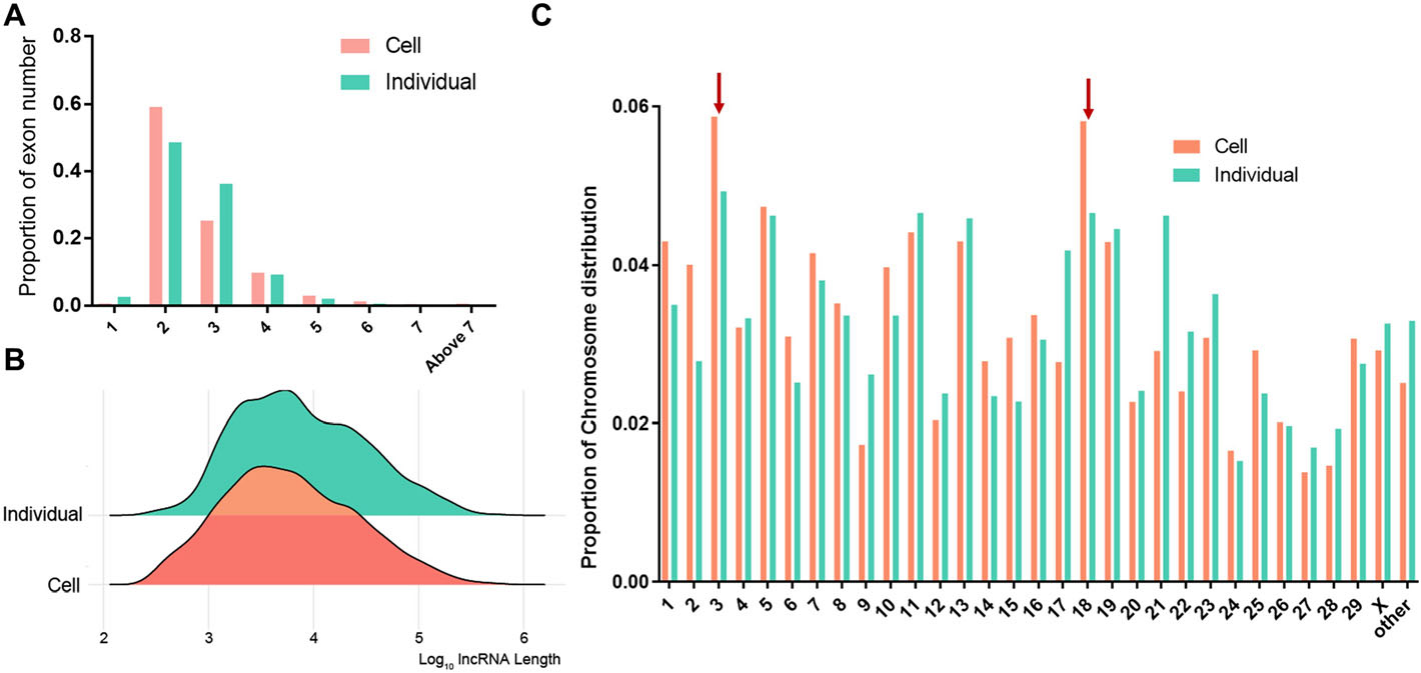
Basic features of lncRNAs obtained in individual and cell samples. (**A**) Number of exons of obtained lncRNA transcripts. (**B**) Length and (**C**) chromosome distributions of obtained lncRNA transcripts. Chromosomes with the highest numbers of lncRNAs are marked with red arrows

Figure 3A–C show the number of exons, length, and chromosome distribution of the lncRNA transcript at the individual and cell levels. More than 90% of identified lncRNA transcript possessed 2–4 exons (Fig. 3A). Approximately 90% of lncRNA transcript length was found to be intensive within 10^3^–10^5^ bp (Fig. 3B). Moreover, these obtained lncRNAs were widely distributed in all chromosomes of the bovine genome, and the highest numbers of obtained lncRNAs existed in chromosomes 3 and 18 (Fig. 3C).

### Identification and functional prediction of differentially expressed lncRNAs

**Fig. 4:**
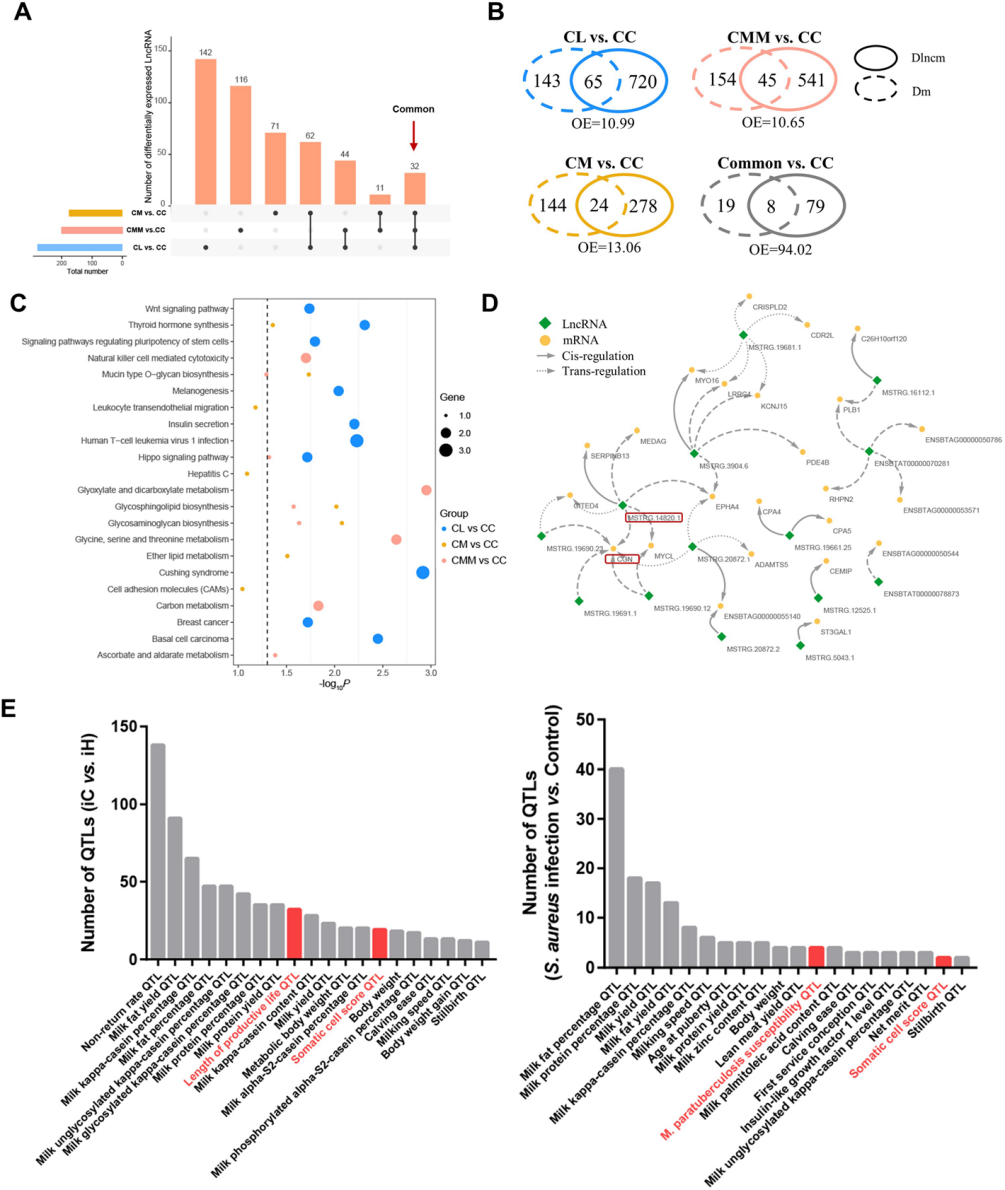
Identification and functional prediction of DE lncRNAs. (**A**) Intersected outcome of DE lncRNAs induced by three different *S. aureus* strain challenge. (**B**) Overlaps between DE mRNA (Dm) and *cis* target mRNA of DE lncRNAs (Dlncm) in cell samples. OE means fold of over-enrichment. (**C**) KEGG enrichment of the *cis* target mRNA of DE lncRNAs. (**D**) Co-expression network of common DE lncRNAs and its *cis/trans* target mRNA. The network was drawn using the Cytoscape software. (**E**) Number of bovine complex traits’ QTLs associated with DE lncRNAs (Top 20 QTLs based on number are shown)

A total of 44, 93, and 100 differentially expressed (DE) lncRNAs were obtained in individual samples, i.e., iC vs. iL, iC vs. iH, and iL vs. iH, respectively, and only three DE lncRNAs were common between iC vs. iL and iC vs. iH (Fig. S1A and Table S1). In cell samples, 280, 176, and 203 DE lncRNAs were identified in CL vs. CC, CM vs. CC, and CMM vs. CC, respectively (Fig. 4A and Table S2), and only 32 DE lncRNAs were common among the three treatments, which indicated the remarkable heterogeneity of host response induced by three different *S. aureus* isolate infections. In addition, the numbers of overlap gene between the *cis* target mRNAs of DE lncRNAs (Dlncm) and actual DE mRNAs (Dm) were assessed. Compared with the expected numbers of overlapped gene, there are 10 times of over-enrichment (OE) observed in the actual numbers of overlapped gene in the cell treatments (Fig. 4B), and 3 times of OE detected in the individual treatments (Fig. S1B). These results indicated that Dm were widely regulated by its neighboring DE lncRNAs.

The functions of the overlapped genes between Dlncm and Dm were annotated by the KEGG pathway. In individual results, Dm were significantly enriched (*P* < 0.05) in some immunity-related pathways, such as *S. aureus* infection, toxoplasmosis, and Toll-like receptor signaling pathway (Fig. S1C). Similar to individual results, the natural killer cell-mediated cytotoxicity, human T-cell leukemia virus 1 infection, and breast cancer could be found in cell samples (Fig. 4C). The interaction network between DE lncRNAs and its *cis*/*trans* target mRNAs in cells is shown in Fig. 4D. Notably, eight DE mRNAs were *cis*/*trans*-regulated by DE lncRNAs MSTRG.14820.1, and Dm *CGN* was widely *trans*-regulated by five DE lncRNAs.

Usually, the genome location of DE lncRNAs with potential function might be close to some quantitative trait locus (QTLs) of animal complex traits. Currently, Cattle QTLdb included 160,659 QTLs representing 675 different complex traits. By comparing the genome location of DE lncRNAs and QTL-associated regions within Cattle QTLdb, in iC vs. iH, 74 out of 93 DE lncRNAs neighbor 1365 QTLs on the genome location, and in the *S. aureus* infection treatment of Mac-T cells vs. control, 23 out of 32 common DE lncRNAs neighbor 195 QTLs (Fig. 4E). The results suggest that these QTLs around DE lncRNAs were associated with milk secretion, health, and reproduction traits. More than 70% of these QTLs were intensive in milk-related traits (Table S3). These QTLs were also associated with immune-related traits, such as *M. paratuberculosis* susceptibility and somatic cell score (a commonly used indicator of mastitis), and reproduction-related traits, such as nonreturn rate and age at puberty.

### Integrated analysis of lncRNAs identified in individual and cell samples

**Fig. 5:**
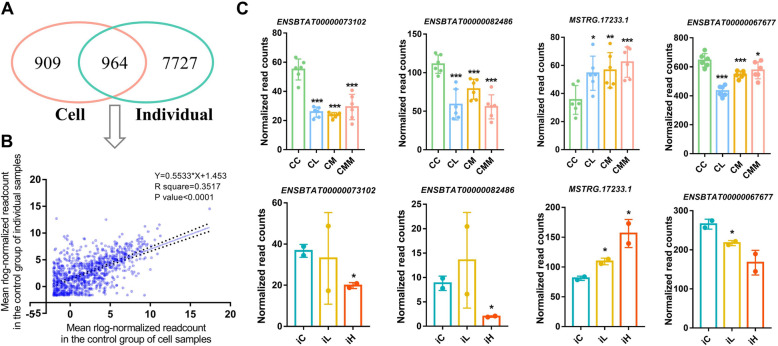
Association of identified lncRNAs between cell and individual samples. (**A**) Venn diagrams for lncRNAs identified in cell and individual control samples. (**B**) Linear regression analysis of the mean rlog-normalized read counts of 964 common lncRNAs in cell and individual control samples. (**C**) Expression level of four stable DE lncRNAs in cell and individual samples with criteria of *P* < 0.05 and same expression change direction

lncRNAs identified in mammary gland tissues of individuals and Mac-T cells were integrated to obtain stable lncRNA markers of mastitis induced by different *S. aureus* strains. A total of 964 lncRNAs were common in individual and cell control samples (Fig. 5A). The linear regression analysis was performed for the mean rlog-normalized read counts of the common 964 lncRNAs. The results (*R*^2^ = 0.3517, *P* < 0.0001; Fig. 5B) showed the significantly moderate correlation between individual and cell samples and indicated that the results outcome of cell samples could represent the partial results outcome of individual samples.

With the criteria of *P* < 0.05 and same expression change direction in individual and cell *S. aureus* infection treatments, four DE lncRNAs, i.e., *ENSBTA00000073102*, *ENSBTA00000082486*, *MSTRG.17233.1*, and *ENSBTA00000067677*, were obtained and defined as stable DE lncRNAs for *S. aureus* infection treatment (Fig. 5C).

The gene sequence-based conservation indicates similar and vital functions across species [19]. For the above four bovine stable DE lncRNAs, the preserved counterparts in humans were sought using the NONCODE (Table 2). Except *ENSBTAT00000082486*, all bovine stable DE lncRNAs had homologous genes in human, and significant somatic cell score QTLs neighbor these lncRNAs within about 20–300 kb. In humans, the preserved counterparts of bovine lncRNA *MSTRG.17233.1* is the progenitor renewal associated non-coding RNA (lncRNA *PRANCR*), which functions as a regulator of epidermal homeostasis [20, 21], and *ENSBTAT00000067677* is the TNK2 antisense RNA 1 (lncRNA *TNK2–AS1*) involved with cell proliferation, migration, and apoptosis [22–24]. Based on their potentially preserved functions, these two lncRNAs were chosen for further analyses. Given their counterparts, i.e., *PRANCR* and *TNK2–AS1* in humans, bovine *MSTRG.17233.1* and *ENSBTAT00000067677* were named as lncRNA *PRANCR* and *TNK2–AS1*, respectively, in this study.

**Table 2:** Information of stable differentially expressed lncRNAs

	Homologous gene in human identified by NONCODE	Gene name	Site information^**a**^	Closest identified QTL^**ba**^
**ENSBTAT00000073102**	NONHSAT003723.2	NA	3:80,327,486-80,347,232	3:80,304,481-80,304,521
**ENSBTAT00000082486**	NA	NA	20:8,025,966-8,027,066	20:7,770,475-7,770,515
**MSTRG.17233.1**	NONHSAT232219.1	***PRANCR***	5:43,266,523-43,268,050	5:43,569,644-43,569,648
**ENSBTAT00000067677**	NONHSAT195289.1	***TNK2-AS1***	1:70,640,306-70,641,382	1:71,741,408-71,949,627

^a^ Assembly: ARS-UCD1.2/bosTau9

^b^Somatic cell score QTL in cow (data from Animal QTL Database)

*QTL*: quantitative trait locus

### lncRNAs *PRANCR* and *TNK2–AS1* as stable markers of *S. aureus* mastitis

**Fig. 6:**
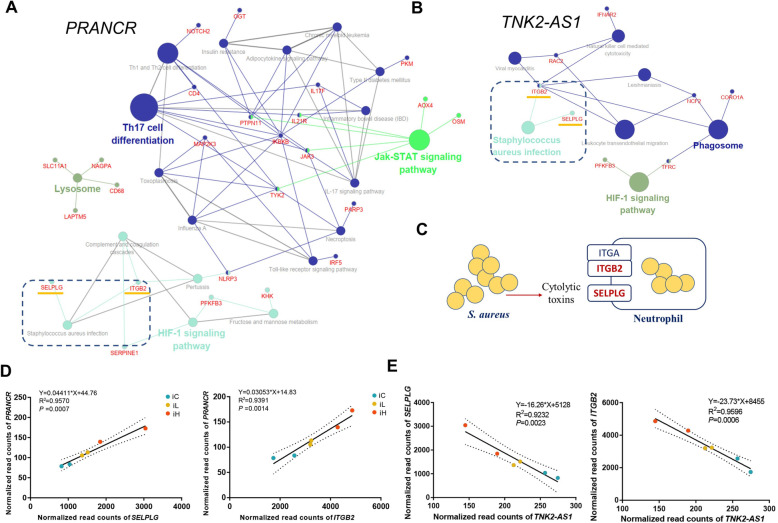
Functional prediction of lncRNAs *PRANCR* and *TNK2–AS1*. KEGG pathway enrichment of *cis* and *trans* target mRNAs of lncRNAs (**A**) *PRANCR* and (**B**) *TNK2–AS1*. (**C**) Part of the genes involved in *S. aureus* infection pathway. Linear regression analysis of normalized read counts among lncRNAs (**D**) *PRANCR*, (**E**) *TNK2–AS1*, and its *trans* target mRNA *SELPLG* and *ITGB2*. *SELPLG*: gene of Selectin P Ligand, *ITGB2*: gene of Integrin Subunit Beta 2

The *trans* and *cis* target mRNAs of lncRNAs *PRANCR* and *TNK2–AS1* were first identified to further understand the potential function of lncRNAs *PRANCR* and *TNK2–AS1*. The KEGG enrichment analysis of these mRNAs showed that amounts of immunity-related pathways, such as *S. aureus* infection, Jak–STAT signaling pathway, Th17 cell differentiation, and phagosome, were enriched (Fig. 6A and B). These results indicated that lncRNAs *PRANCR* and *TN2–AS1* were widely involved in the inflammatory response induced by *S. aureus* challenge. Integrin subunit beta 2 (ITGB2) and selectin P ligand (SELPLG) within the pathway of *S. aureus* infection were the receptors of *S. aureus* cytolytic toxins (Fig. 6C), and the results of linear regression analysis showed that the bovine milk somatic cell count (SCC) and the expression of *ITGB2* and *SELPLG* were positively correlated with lncRNA *PRANCR* and negatively correlated with lncRNA *TNK2–AS1* (*R*^2^ > 0.92, *P* < 0.01, Figs. S2A, 6D, and 6E). In addition, a significantly negative expression correlation was observed between *PRANCR* and *TNK2–AS1* (Fig. S2B).

### Folic acid protect host against *S. aureus* challenge by regulating the expression level of lncRNAs involved in toxin transport and inflammatory response-related pathways

**Fig. 7:**
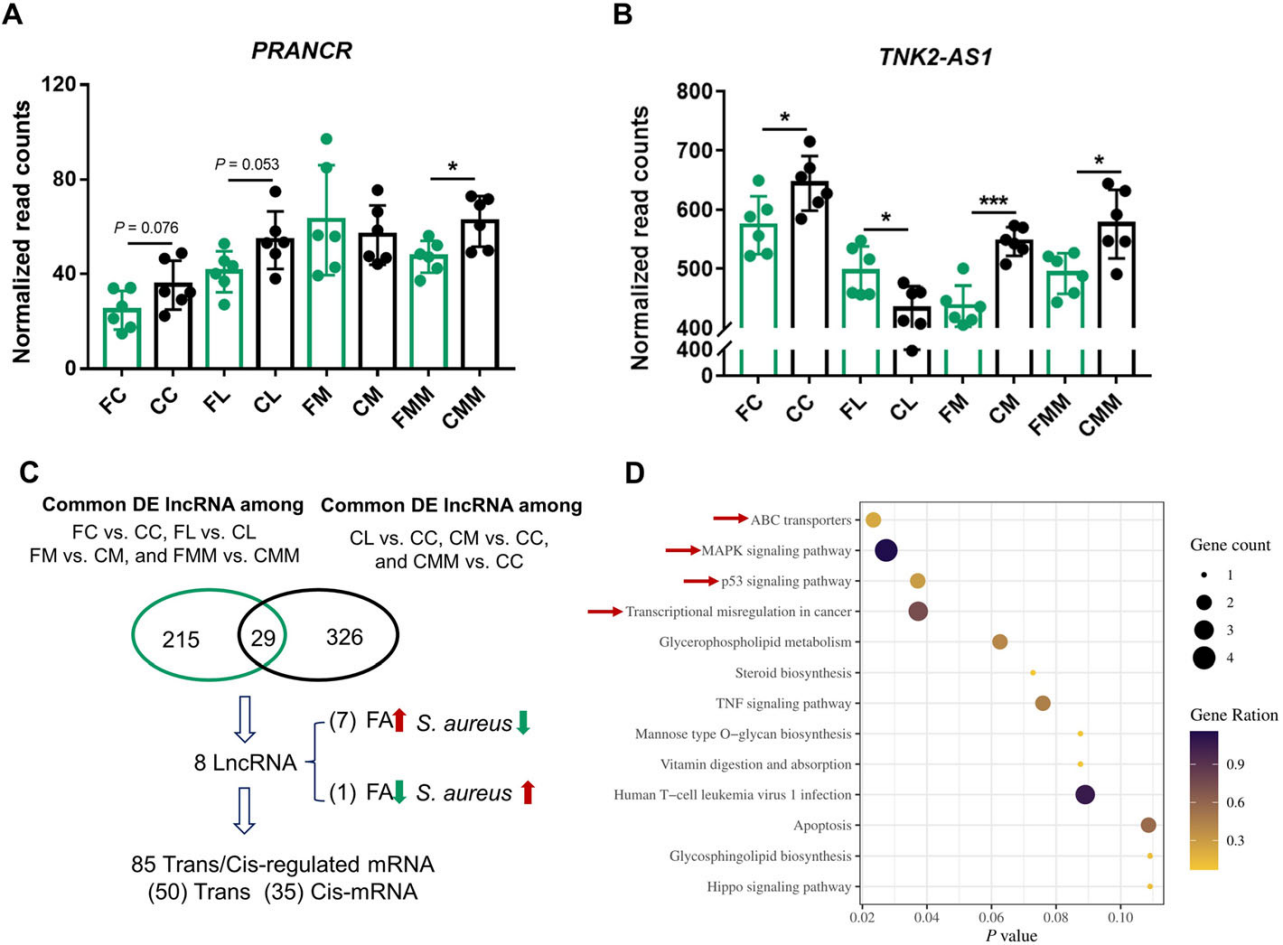
*Cis* and *trans* target mRNAs of key lncRNAs influenced by folic acid that are involved in toxin transport and inflammatory response-related pathways. Normalized read counts of lncRNAs (**A**) *PRANCR* and (**B**) *TNK2–AS1* in different groups. (**C**) Intersected results and *cis*/*trans* target mRNA identification of lncRNAs between folic acid and *S. aureus* treatment groups. Here, DE lncRNAs were defined with the criteria of *P* < 0.05. (**D**) KEGG enrichment results of the target mRNAs of the eight key lncRNAs

FC, FL, FM, and FMM groups were established to understand the interplays among host lncRNAs, the target mRNAs of lncRNA, *S. aureus* infection, and FA treatment (Fig. 1). First, the effects of FA on lncRNAs *PRANCR* and *TNK2–AS1* were investigated. Regarded to *PRANCR*, FA can hamper the upregulation of the lncRNA in the comparisons of CC vs. FC, CL vs. FL, and CMM vs. FMM (Fig. 7A). As for *TNK2–AS1*, the downregulation of the lncRNA is hampered by FA only in the comparison of CL vs. FL (dotted box in Fig. 7B). The preliminary results indicated the regulatory roles of FA in inflammation response induced by specific *S. aureus* strain challenge.

The relationship between DE lncRNAs induced by *S. aureus* infection or FA treatment was further investigated. A total of 244 common DE lncRNAs in the comparisons between FA treatment and corresponding control (i.e., FC vs. CC, FL vs. CL, FM vs. CM, and FMM vs. CMM) and 355 common DE lncRNAs in the comparisons between *S. aureus* infection and control (CL vs. CC, CM vs. CC, and CMM vs. CC) were obtained, and 29 DE lncRNAs intersected between the above 244 and 355 common DE lncRNAs. In 8 of these 29 lncRNAs, the expression changes induced by *S. aureus* infection could be reduced and reversed by FA treatment (Fig. 7C). Then, 50 *trans* and 35 *cis* target mRNAs of these eight lncRNAs were obtained. The results of KEGG enrichment showed that the toxin transport and inflammatory response-related pathways (e.g., ABC transporters, MAPK signaling pathway, and p53 signaling pathway) were regulated by these lncRNAs (Fig. 7D).

### lncRNAs *PRANCR* and *TNK2–AS1* were associated with hematological parameters

**Fig. 8:**
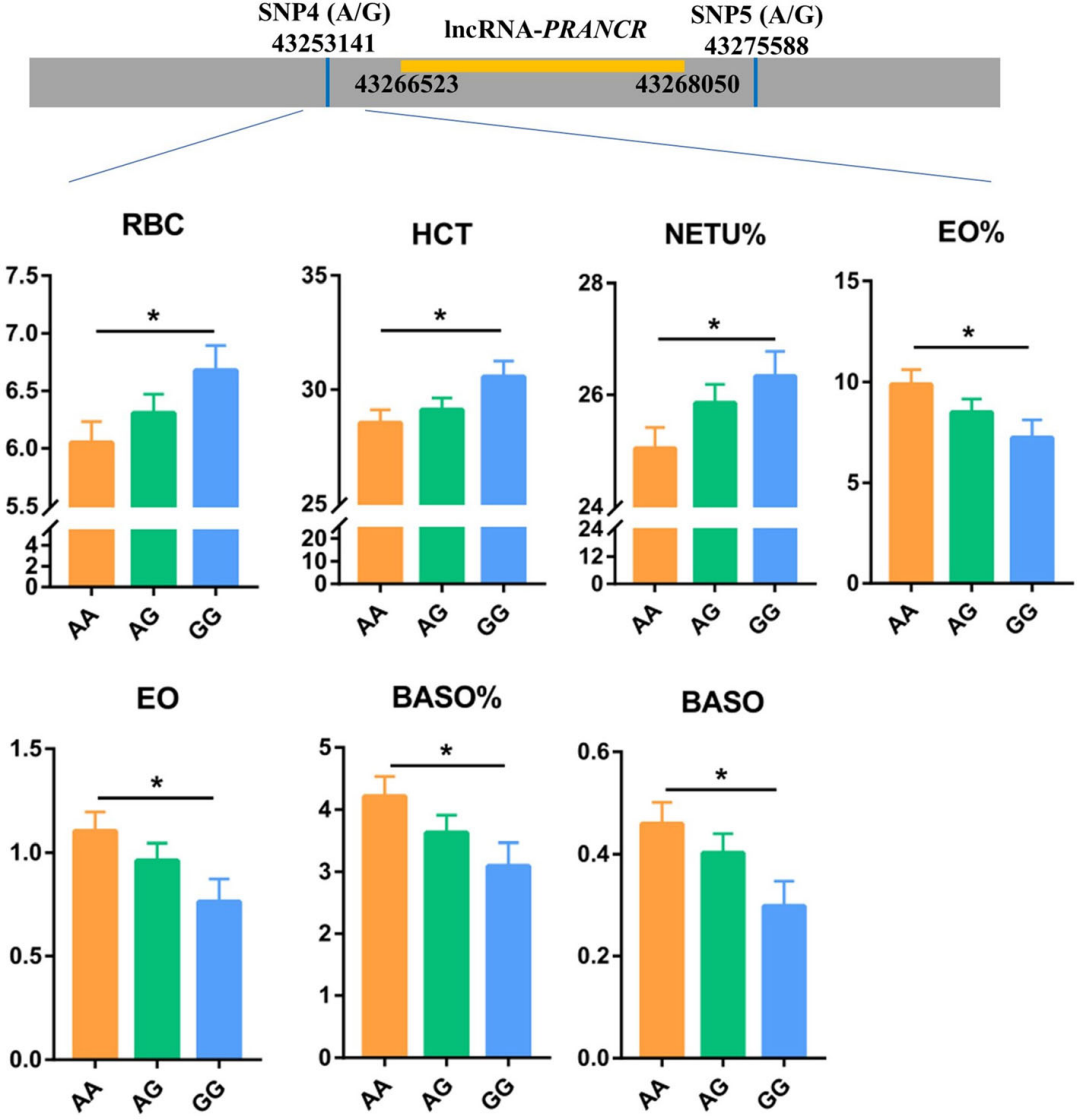
Blood routine test parameters significantly associated with the SNP4 of lncRNA *PRANCR*. The grey box notes the fragment of chromosome, the yellow box notes the location of lncRNA *PRANCR*. RBC: Red blood cell; HCT: Red blood cell specific volume; NETU%: Neutrophils ratio; EO%: Eosinophil ratio; EO#: Eosinophil counts; BASO%: Basophile ratio; BASO#: Basophile counts

The potential association between SNPs within 100 kb lncRNAs *PRANCR* and *TNK2–AS1* and hematological parameters (HPs) were analyzed to further validate the function of lncRNAs *PRANCR* and *TNK2–AS1* on immune response (Table S4). For the lncRNA *PRANCR*, 4 out of 11 SNPs were significantly associated with the partial indicators of HP (Table S5, *P* < 0.05). Significant associations between SNP4 and red blood cell (RBC), red blood cell specific volume (HCT), percentage of neutrophil (NETU%), percent of eosinophile granulocyte count (EO%), eosinophile granulocyte count (EO#), percent of basophilic granulocyte count (BASO%), and basophilic granulocyte count (BASO#, Fig. 8), and between SNP8 and mean corpuscular hemoglobin (MCH) were found. Moreover, SNP10 and SNP11 were significantly associated with mean corpuscular volume (MCV). Among the 11 SNPs, SNP4 was the closest to the location of the lncRNA *PRANCR*. Furthermore, the NETU%, EO#, EO%, BASO#, and BASO% were key indicators reflecting the host immune level. Similar to the lncRNA *PRANCR*, a nearly significant association existed between the SNP closest to the lncRNA *TNK2–AS1* and the platelet-large cell ratio (P-LCR) of HP (Table S6 and Fig. S3). These results showed that lncRNAs *PRANCR* and *TNK2–AS1* could be regarded as indicators of cow immune status.

### lncRNA *PRANCR* influences the apoptosis of Mac-T cells induced by *S. aureus* challenge

**Fig. 9:**
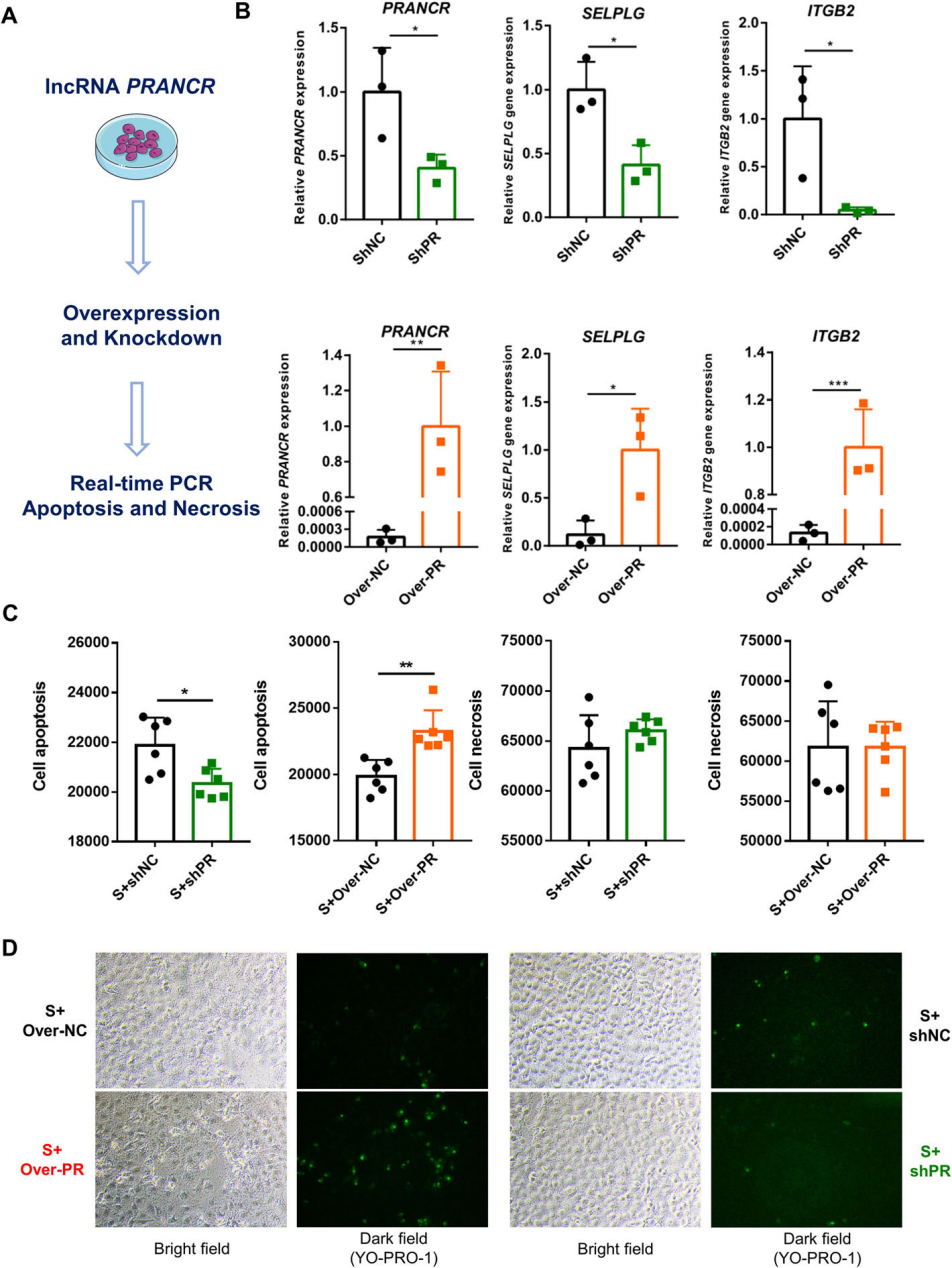
Involvement of *PRANCR* with *S. aureus* infection pathway and the regulation of cell apoptosis. (**A**) Workflow of *PRANCR* function validation. (**B**) Relative expression levels of *PRANCR*, *SELPLG*, and *ITGB2*. (**C**) Functions of *PRANCR* on cell apoptosis and necrosis. (**D**) Cell apoptosis was assessed with fluorescence microscopy. Apoptotic cells were stained green by YO-PRO-1. S: *S. aureus* challenge, Over: plasmid for lncRNA overexpression, sh: the shRNA for lncRNA knockdown, NC: negative control, and PR: lncRNA *PRANCR*

Finally, lncRNA *PRANCR* was chosen for the experiment of gene knockdown and overexpression to confirm the function of the lncRNA on regulating the cell immune response (Fig. 9A). First, the regulation of *PRANCR* on its trans target mRNAs was investigated. Consistent with the results of linear regression (Fig. 6D), the knockdown of lncRNA *PRANCR* significantly reduced the mRNA expression of *SELPLG* and *ITGB2*, and the overexpression of this lncRNA significantly increase the mRNA expression of *SELPLG* and *ITGB2* (*P* < 0.05, Fig. 9B). Subsequently, the regulatory effects of lncRNA *PRANCR* on cell apoptosis and necrosis were investigated. Our results showed the knockdown or overexpression of lncRNA *PRANCR* doesn’t influence cell apoptosis and necrosis (Fig. S4). It is worth noted that the knockdown of lncRNA *PRANCR* remarkably reduced the cell apoptosis induced by *S. aureus* challenge, and that the overexpression of this lncRNA significantly promoted the cell apoptosis, and this lncRNA cannot influence cell necrosis (Fig. 9C). The results of fluorescence staining also verified the function of lncRNA *PRANCR* on cell apoptosis induced by *S. aureus* challenge (Fig. 9D).

## Discussion

Increasing evidence reveals that lncRNAs are widely involved in host response to pathogen invasion [25–27]. In this study, the basic features of obtained lncRNA transcripts were first characterized in Mac-T cells *in vitro* and bovine mammary gland tissue samples *in vivo*. Owing to the bigger data size and higher mapping rate in cell samples than in individual samples (Table 1), more lncRNA transcripts were identified in cell samples (Fig. 2A). Most identified lncRNA transcripts were located in intergenic regions, and this finding was consistent with those in previous studies [28–30]. Moreover, our results showed that lncRNAs were widely distributed on all chromosomes especially the largest number of Chromosome 3, and this finding was similar to that of previous studies on Mac-T cells [31]. The results indicated the potentially extensive involvement of lncRNAs in bovine complex traits.

*S. aureus* is one of the main pathogens of bovine mastitis [3, 32]. Previous studies reported that several bovine lncRNAs may regulate the host immune response to *S. aureus* infection. In the cell model of bovine *S. aureus* mastitis, the *S. aureus* adhesion to epithelial cells can be mediated by lncRNAs *TUB* and *H19* [31, 33], and the NF-κB/NLRP3 inflammasome pathway is regulated by the lncRNA *XIST* [34]. However, the host immune response to *S. aureus* is demonstrated to be dependent on the lineages of this bacterium [7, 32, 35]. Until now, the heterogeneity and similarity of lncRNAs involved in inflammatory response induced by different *S. aureus* strain infection are not explained well. In our analysis, only 32 DE lncRNAs were shared among the three different bovine milk-originated *S. aureus* strains, which reminded us that when studying host–pathogen interactions, heterogeneity between strains should be considered. The chronic inflammation engaged in the progress of cancer is widely recognized [36–38], and the pathways of breast cancer and basel cell carcinoma are exclusively enriched in the comparison of CL vs. CC, which implied the potential harm of strain L (isolated from a cow with low milk SCC) to the host. Moreover, previous studies reported that the host’s inflammatory response and cytokine expression level are dependent on pathogen concentration [39–41]. Our results also showed few overlaps of DE lncRNAs (only 3 common DE lncRNAs) and differentially activated KEGG pathways between the comparisons of iC vs. iL and iC vs. iH. Thus, identifying the stable molecular markers of *S. aureus* mastitis is difficult under different *S. aureus* lineages and loads.

Emerging evidence indicates that many lncRNAs act their functions by interacting with mRNA in *cis*- and *trans*-manner [42–45]. In the present study, the significant overlaps between *cis* target Dlncms and Dms also confirmed that the differential expression of mRNA could be regulated by its neighboring DE lncRNAs. Previous studies identified numbers of lncRNAs located in the QTLs of complex traits among several species [46–49]. The results of the present study showed that 4/23 (17.39%) and 25/74 (33.78%) DE lncRNAs were associated with health-related QTLs in CC vs. *S. aureus* infection groups (CL, CM, and CMM) and in iC vs. iH (Table S3), respectively. Moreover, 17/23 (73.91%) and 59/74 (79.73%) DE lncRNAs in CC vs. *S. aureus* infection groups and iC vs. iH, respectively, were related to multi-trait QTLs, which implied the multiple effects of these lncRNAs on the regulation of milk production and immunity-related traits.

Immortalized Mac-T cells are primarily established from primary bovine mammary alveolar cells [50]. In this study, the tissue punch biopsy was adopted, and the samples of obtained mammary gland consisted of several tissues and cells, such as mammary epithelial cell, adipose tissue and connective tissue. Besides, neutrophils also widely exist in the mammary gland after infected with pathogen [51, 52]. The above differences between Mac-T cells and mammary gland tissues may explain the few shared DE lncRNAs between cell and individual samples (Fig. 5A) in the current study. The significantly moderate correlation (0.3517) of lncRNA expression between Mac-T cells and mammary gland tissues indicated that Mac-T cells cannot be completely regarded as the alternative of bovine mammary gland. The results of Mac-T cells should be tested at the bovine individual level.

Under the different *S. aureus* strains challenge, the consistent expression changes of four lncRNAs were observed at the cell and individual levels. Among these four lncRNAs, three lncRNAs were conserved between human and cattle. Evidence shows that lncRNA *TNK2–AS1* downregulation can inhibit cell proliferation and migration and promote apoptosis [22–24]. Moreover, in human, lncRNA *PRANCR* is closely related with epidermis formation and ovarian cancer metastasis [20, 21, 53]. Our study found the regulatory effect of *PRANCR* on the cell apoptosis induced by *S. aureus* challenge. In addition, the regulation of the lncRNA *PRANCR* on the mRNA expression of *SELPLG* and *ITGB2* within *S. aureus* infection pathway were confirmed in the overexpression and knockdown validation. However, whether the regulatory effect of the lncRNA on cell apoptosis by directly mediating *SELPLG* and *ITGB2* remains to be further studied. The HP detection can help diagnose organ and systemic disorders in dairy cow [54–56]. The significant associations between SNPs closest to these two lncRNAs and partial HP indicated that SNPs nearby *PRANCR* and *TNK2–AS1* could be regarded as potential molecular markers affecting the immunity of dairy cows. In other studies, the association between SNPs and HP is also widely investigated [57, 58], but the causal mutation within these two lncRNAs should be further explored for future animal breeding.

A healthy mammary gland with immune equilibrium is essential for the host to fight against pathogen infection [7]. Previous studies reported that moderately extra FA intake can improve the host immune capacity and reduce inflammation and oxidative stress [59–61]. Our previous results also indicated that for dairy cows, FA supplementation reduces mastitis incidence and promotes milk production [4, 5]. In Mac-T cells infected with *S. aureus*, the results of the present study showed that the expression of genes within ABC transporters [62] involved with toxin secretion, MAPK signaling pathway [63] and p53 signaling pathway [64] related with inflammatory regulation can be influenced by FA treatment through the regulation of lncRNAs. FA is a kind of methyl-donor. A previous study found that methyl-donor supplemented diet prevents the colonization of intestinal *E. coli* in a mouse model of Crohn’s disease, which may be by influencing the DNA methylation level of *CEACAM6* gene (a kind of cell adhesion molecule) [65]. Similarly, in mice, FA supplementation prevent Helicobacter-associated gastric cancer by increasing global DNA methylation [13]. Thus, in the present study, whether FA affects lncRNA expression by affecting DNA methylation needs further exploration. Our findings will help understand the roles of lncRNA in *S. aureus* infection and provide an improved approach for the effective diagnosis and prevention of bovine *S. aureus* mastitis.

## Conclusion

**Fig. 10:**
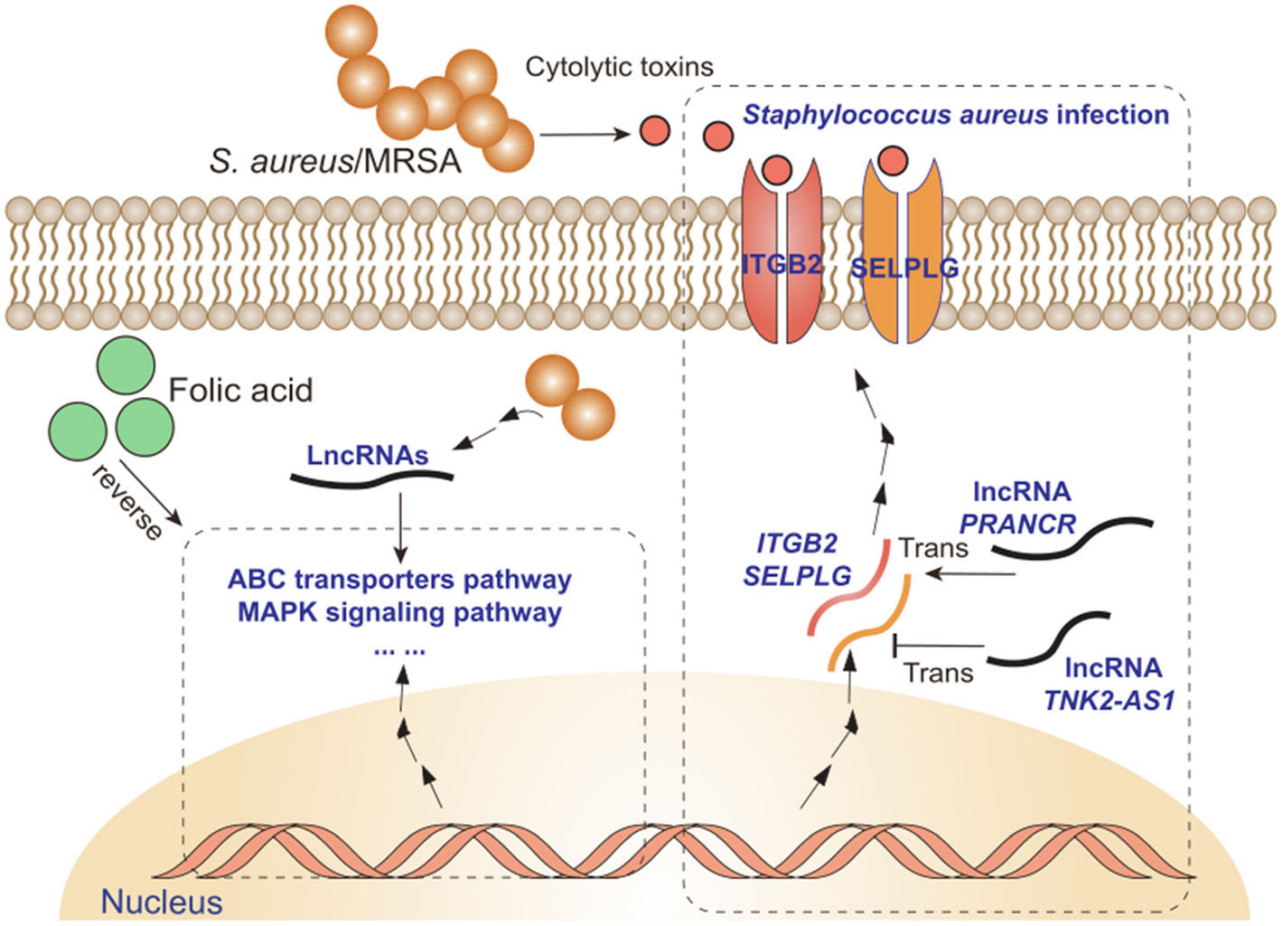
Interplays among host immune responses of transcriptome (lncRNA and mRNA), *S. aureus* infection, and folic acid treatment

Consequently, this study characterized an important lncRNA resource of the interplays among bovine mammary gland tissues/Mac-T cells, different *S. aureus* strains infection, and FA treatment (Fig. 10). lncRNAs *PRANCR* and *TNK2–AS1* can be regarded as stable molecular markers of bovine *S. aureus* mastitis. The negative effect of *S. aureus* infection on the host can be partially prevented and reduced by FA supplementation.

## Supplementary Information

**Additional file 1: Figure S1.** Identification and KEGG enrichment of *cis* target mRNA of DE lncRNAs. (A) Venn diagrams among the different comparisons of individual samples. (B) Overlaps between DE mRNA and *cis* target mRNA of DE lncRNAs in individual samples. OE means fold of over-enrichment. (C) KEGG enrichment of *cis* target mRNA of DE lncRNAs. iC: mammary gland challenged with saline; iL: mammary challenged with low concentration of *S. aureus*; iH: mammary challenged with high concentration of *S. aureus*. **Figure S2.** (A) Correlations between somatic cell count and lncRNAs *PRANCR* and *TNK2–AS1*. (B) Correlation between the two lncRNAs. **Figure S3.** Blood routine test parameters significantly associated with the SNP4 of lncRNA *TNK2-AS1*. The grey box notes the fragment of chromosome, the green box notes the location of lncRNA *TNK2-AS1*. P-LCR: Platelet-large cell ratio; RBC: Red blood cell. **Figure S4.** The influence of *PRANCR* on cell apoptosis and necrosis. Over: plasmid for lncRNA overexpression, sh: the shRNA for lncRNA knockdown, NC: the negative control, and PR: lncRNA *PRANCR*.

**Additional file 2. **DE lncRNAs *in vivo*.

**Additional file 3. **DE lncRNAs *in vitro.*

**Additional file 4. **Bovine QTL information

**Additional file 5. **SNP information

**Additional file 6.** Association between *PRANCR* SNPs and HPs

**Additional file 7. Association between ***TNK2-AS1* SNPs and HPs

## Data Availability

RNA-seq data (*in vivo*) had been submitted to the NCBI’s Sequence Read Archive (SRA) with the accession number SRP073432. RNA-Seq (*in vitro*) and 150 K SNP-chip data from China Agricultural University is available upon the agreement of China Agricultural University and should be requested directly from the authors.

## Abbreviations

*S. aureus*: *Staphylococcus aureus*
MRSA: Methicillin-resistant *S. aureus*
SCC: Somatic cell count
FA: Folic acid
Strain L: *S. aureus* isolated from the cow with low milk somatic cell counts
Strain M: *S. aureus* isolated from the cow with mastitis
Strain MM: MRSA isolated from the cow with mastitis
Mac-T cells: Mammary alveolar cells
CC: Control + control *in vitro*
CL: Control + Strain L infection *in vitro*
CM: Control + Strain M infection *in vitro*
CMM: Control + Strain MM infection *in vitro*
FC: Folic acid treatment + control *in vitro*
FL: Folic acid treatment + Strain L infection *in vitro*
FM: Folic acid treatment + Strain M infection *in vitro*
FMM: Folic acid treatment + Strain MM infection *in vitro*
iC: Individual Control
iL: Individual challenged with Low concentration of *S. aureus*
iH: Individual challenged with High concentration of *S. aureus*
DE: Differentially expressed
lncRNA: Long non-coding RNA
ilncRNA: Intronic lncRNA
lincRNA: Intervening lncRNA
Dlncm: *Cis* target mRNAs of differentially expressed lncRNA
Dm: Differentially expressed mRNA
lncNAT: Antisense lncRNA
*PRANCR*: Progenitor renewal associated non-coding RNA
*TNK2-AS1*: TNK2 antisense RNA 1
HP: Hematological parameters

## And the abbreviations of hematological parameters are shown as below

WBC: White blood cell
RBC: Red blood cell
HGB: Haemoglobin
HCT: Red blood cell specific volume
MCV: Mean Corpuscular Volume
MCH: Mean corpuscular hemoglobin
MCHC: Mean corpuscular hemoglobin concentration
PLT: Platelet count
NETU%: Neutrophils ratio
NETU#: Neutrophil counts
LYMPH%: Lymphocyte ratio
LYMPH#: Lymphocyte counts
MONO%: Monocyte ratio
MONO#: Monocyte counts.
EO%: Eosinophil ratio
EO#: Eosinophil counts
BASO%: Basophile ratio
BASO#: Basophile counts
PDW: Platelet distribution width
MPV: Mean platelet volume
RDW-SD: Red cell distribution width-stand error
RDW-CV: Red cell distribution width-coefficient of variation
P-LCR: Platelet-large cell ratio
PCT: Platelet cubic measure distributing width.
