# Supplementary figures and images for: Transcriptome sequencing analysis for the identification of stable lncRNAs associated with bovine Staphylococcus aureus mastitis
Source: J Anim Sci Biotechnol. 2021 Dec 13;12:120. doi: 10.1186/s40104-021-00639-2 (PMC8667444; doi:10.1186/s40104-021-00639-2)

**Figure S1**


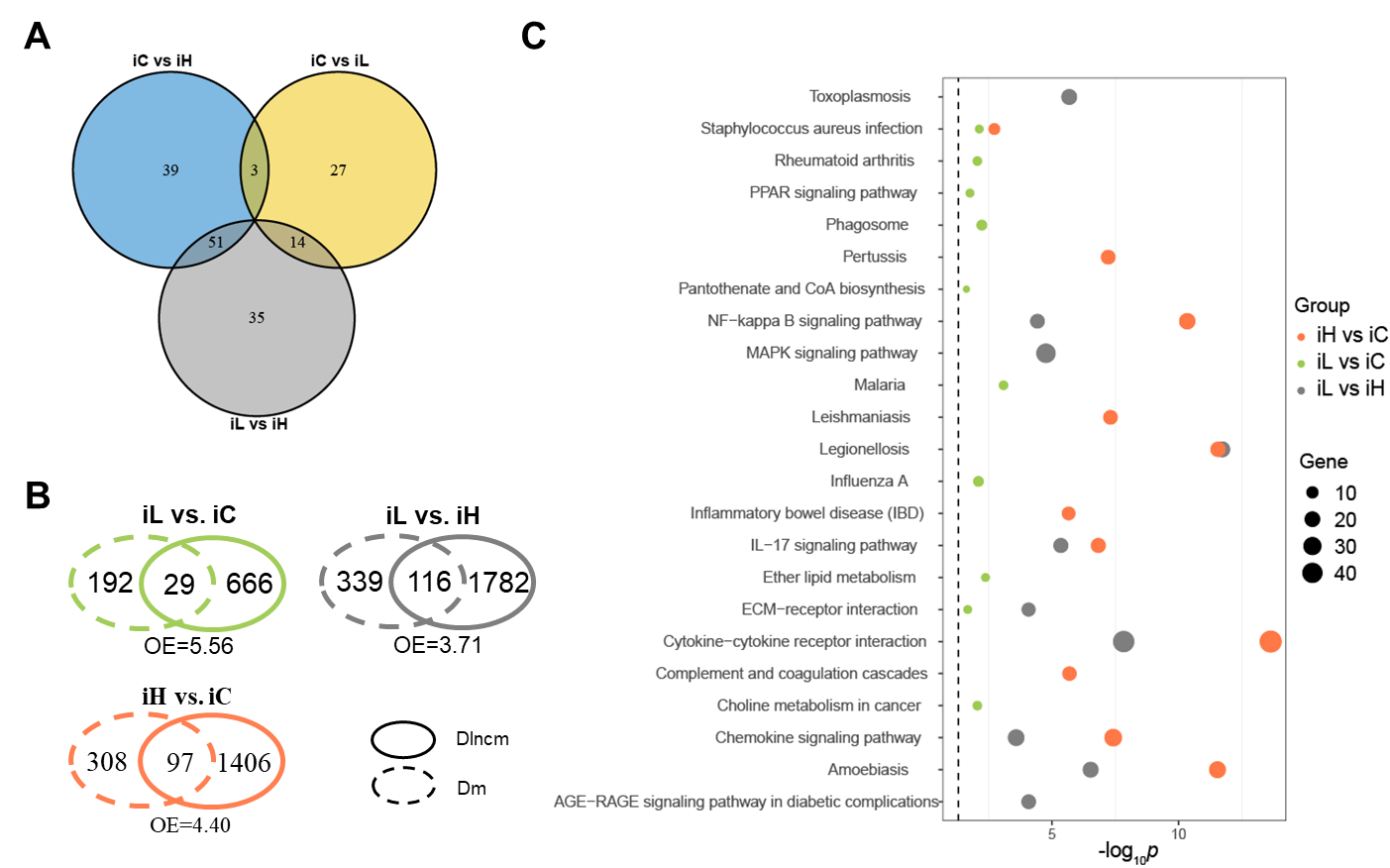


**Figure S2**


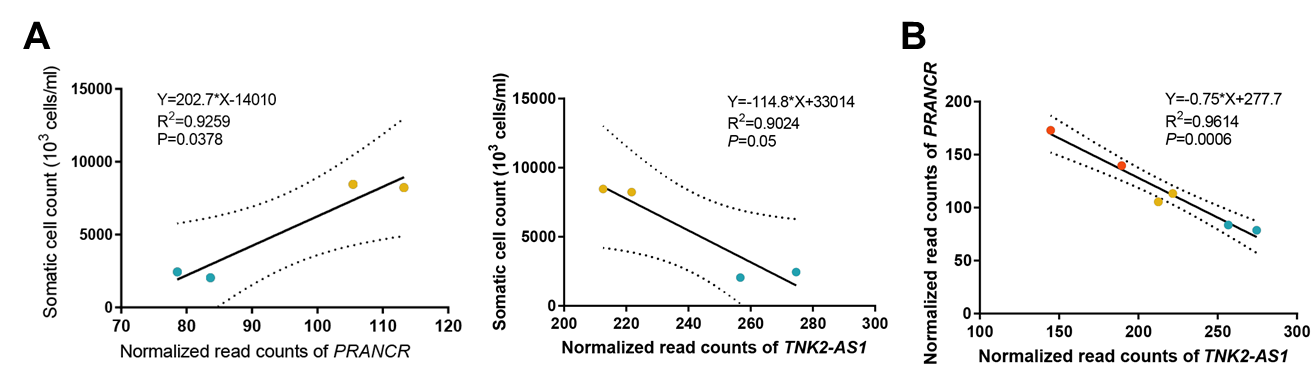


**Figure S3**


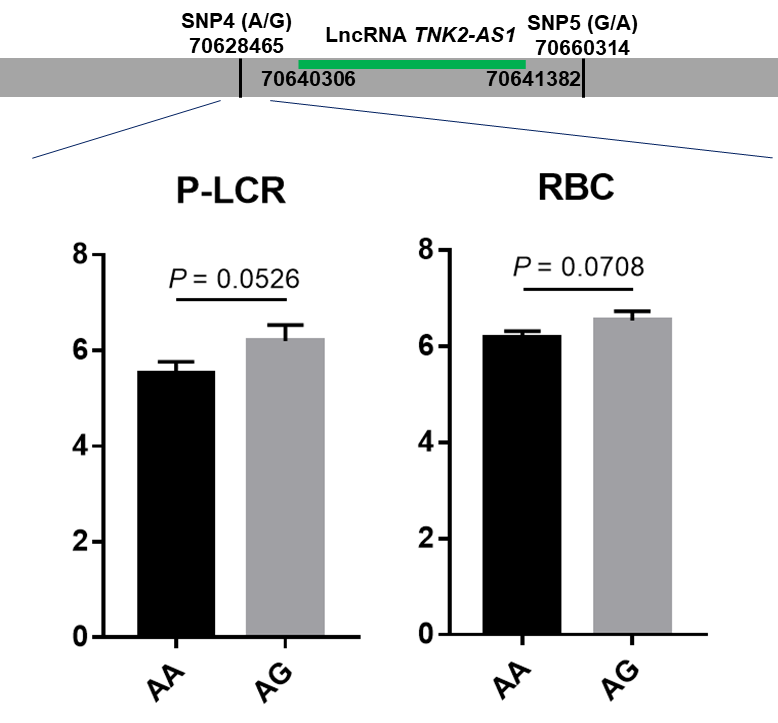


**Figure S4**


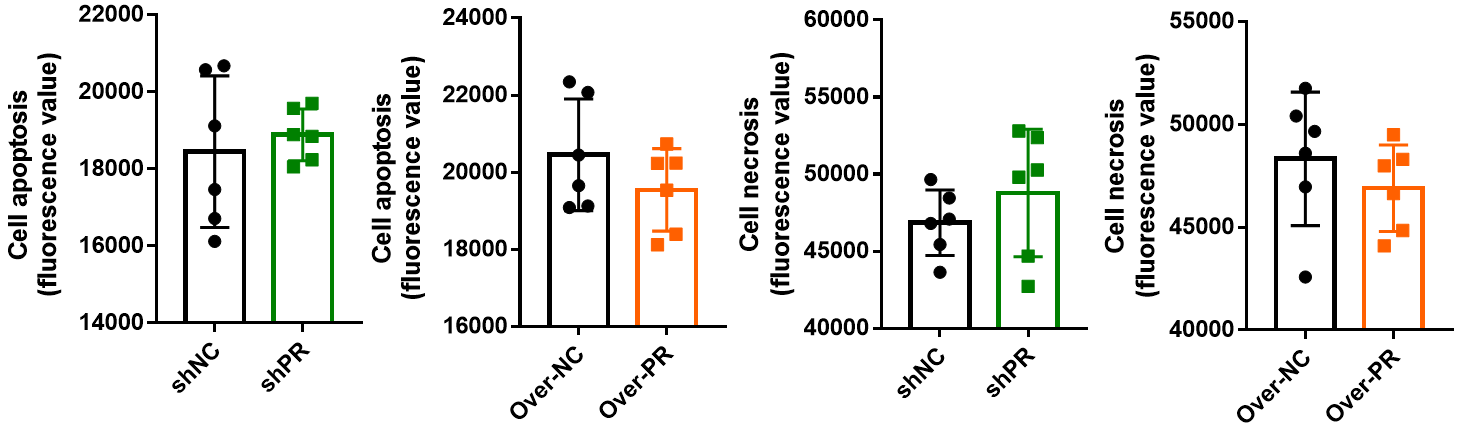

Supplement: Supplementary file 1 — Additional file 1: Figure S1. Identification and KEGG enrichment of cis target mRNA of DE lncRNAs. (A) Venn diagrams among the different comparisons of individual samples. (B) Overlaps between DE mRNA and cis target mRNA of DE lncRNAs in individual samples. OE means fold of over-enrichment. (C) KEGG enrichment of cis target mRNA of DE lncRNAs. iC: mammary gland challenged with saline; iL: mammary challenged with low concentration of S. aureus; iH: mammary challenged with high concentration of S. aureus. Figure S2. (A) Correlations between somatic cell count and lncRNAs PRANCR and TNK2–AS1. (B) Correlation between the two lncRNAs. Figure S3. Blood routine test parameters significantly associated with the SNP4 of lncRNA TNK2-AS1. The grey box notes the fragment of chromosome, the green box notes the location of lncRNA TNK2-AS1. P-LCR: Platelet-large cell ratio; RBC: Red blood cell. Figure S4. The influence of PRANCR on cell apoptosis and necrosis. Over: plasmid for lncRNA overexpression, sh: the shRNA for lncRNA knockdown, NC: the negative control, and PR: lncRNA PRANCR. [file 40104_2021_639_MOESM1_ESM.docx]
